# Stereoselectivity in the Membrane Transport of Phenylethylamine Derivatives by Human Monoamine Transporters and Organic Cation Transporters 1, 2, and 3

**DOI:** 10.3390/biom12101507

**Published:** 2022-10-18

**Authors:** Lukas Gebauer, Muhammad Rafehi, Jürgen Brockmöller

**Affiliations:** Institute of Clinical Pharmacology, University Medical Center Göttingen, D-37075 Göttingen, Germany

**Keywords:** monoamine transporters, organic cation transporters, stereoselective drug transport, chiral HPLC, phenylethylamines, neurotransmitter transport

## Abstract

Stereoselectivity is well known and very pronounced in drug metabolism and receptor binding. However, much less is known about stereoselectivity in drug membrane transport. Here, we characterized the stereoselective cell uptake of chiral phenylethylamine derivatives by human monoamine transporters (NET, DAT, and SERT) and organic cation transporters (OCT1, OCT2, and OCT3). Stereoselectivity differed extensively between closely related transporters. High-affinity monoamine transporters (MATs) showed up to 2.4-fold stereoselective uptake of norepinephrine and epinephrine as well as of numerous analogs. While NET and DAT preferentially transported *(S)*-norepinephrine, SERT preferred the *(R)*-enantiomer. In contrast, NET and DAT showed higher transport for *(R)*-epinephrine and SERT for *(S)*-epinephrine. Generally, MAT stereoselectivity was lower than expected from their high affinity to several catecholamines and from the high stereoselectivity of some inhibitors used as antidepressants. Additionally, the OCTs differed strongly in their stereoselectivity. While OCT1 showed almost no stereoselective uptake, OCT2 was characterized by a roughly 2-fold preference for most *(R)*-enantiomers of the phenylethylamines. In contrast, OCT3 transported norphenylephrine and phenylephrine with 3.9-fold and 3.3-fold preference for their *(R)*-enantiomers, respectively, while the para-hydroxylated octopamine and synephrine showed no stereoselective OCT3 transport. Altogether, our data demonstrate that stereoselectivity is highly transporter-to-substrate specific and highly diverse even between homologous transporters.

## 1. Introduction

Adrenergic neurotransmitters and their derivatives control most important vegetative functions but also higher brain functions such as memory, emotion, and cognition [1]. Besides the major catecholamine neurotransmitters dopamine, epinephrine, and norepinephrine, other phenylethylamines may also act as neurotransmitters, and several of them are classified as trace amines [2,3]. Catecholamines exert their functions mainly by binding to extracellular adrenergic receptors [4]. Stereoselectivity in the activity of epinephrine was already discovered more than a century ago, and the schematic three-point model of the interaction of *(R)*-(−)-epinephrine to its receptor is presented in many pharmacology textbooks (e.g., [5]). However, stereoselectivity in the pharmacokinetics of epinephrine and norepinephrine is much less well studied. Additionally, several trace amines bind intracellularly to trace-amine associated receptors [2]. Thus, stereoselective cell uptake of these substances is not only relevant for their elimination but also for some of their not yet fully understood physiological functions.

**Figure 1:**
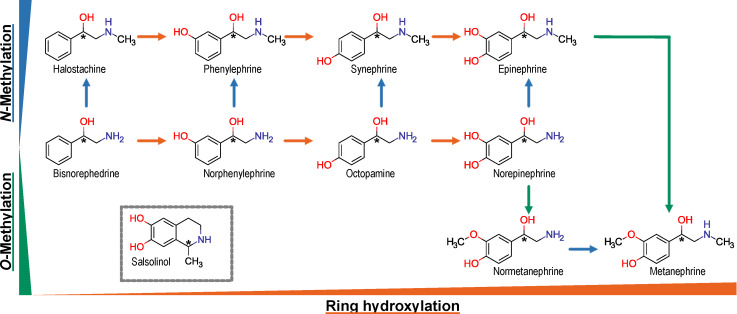
Chemical relationship of investigated substances. Apart from salsolinol, all substances were beta-hydroxylated phenylethylamines. All substances differed only by single structural alterations, including hydroxylation of the phenolic ring, methylation of the primary amino group and methylation of the para-hydroxy group. Salsolinol is characterized by a second ring which may be formed by condensation of dopamine with acetaldehyde. Chiral centers are marked with an asterix (*).

The phenylethylamines investigated here include derivatives with only a single phenolic hydroxyl group or completely lacking phenolic ring hydroxylation (Figure 1). Phenylethylamines are derived from the aromatic amino acids phenylalanine and tyrosine. Bisnorephedrine is the most simple chiral phenylethylamine. It is biosynthesized from phenylethylamine by the dopamine-β-hydroxylase (DBH) [6]. Norphenylephrine and Octopamine, two single ring-hydroxylated phenylethylamines, are biosynthesized by DBH from meta- and para-tyramine, respectively [7,8]. Norepinephrine is biosynthesized from dopamine by DBH [9]. The *N*-methylated analogues are usually formed by action of the phenylethanolamine-*N*-methyl transferase [7]. Salsolinol may arise endogenously from the condensation of dopamine and acetaldehyde [10].

All phenylethylamines have a single amino group positively charged at physiological pH [11]; thus, passive nonionic diffusion over cellular membranes is limited. Several transporter proteins facilitate the uptake of phenylethylamines. Predominantly, high-affinity monoamine transporters (MATs) of the SLC6 gene family control synaptic levels of monoamine neurotransmitters mostly by presynaptic reuptake of released neurotransmitters [12]. The norepinephrine (NET/*SLC6A2*), dopamine (DAT/*SLC6A3*), and serotonin transporters (SERT/*SLC6A4*) are characterized by a high affinity towards their name-giving substrates. However, they also transport the other monoamine neurotransmitters and various other substrates as well [13,14]. MATs are mainly but not exclusively expressed in specific areas of the brain [15]. In addition, the SLC22 organic cation transporters (OCT1, -2, and -3/*SLC22A1*, *2*, and *3*) include most monoamines as their substrates [16]. OCTs are polyspecific transporters involved in the cellular uptake of many drugs and certain endogenous compounds [17,18]. OCT1 and OCT2 are mainly expressed in the liver and kidney [19], respectively, whereas OCT3 is more broadly expressed. Not only the MATs but also OCT2 and OCT3 are expressed in the brain [20,21], and OCT3 also appears to be located at the blood–brain barrier [22]. Initially, OCTs of the SLC22 family have been considered to be mainly responsible for the monoamine clearance in peripheral organs [23], but novel data indicate that OCTs are also directly involved in monoaminergic signaling in the brain [20,24,25,26,27].

Stereoselectivity is a well-known phenomenon in drug metabolism and receptor binding. However, only a few studies have addressed stereoselectivity in drug membrane transport [28,29], although an unequal transport might be highly relevant for the pharmacokinetics and toxicokinetics of chiral molecules. For OCTs, the stereoselective uptake of selected adrenergic drugs has been studied previously [30,31]. The degree of stereoselectivity differed between closely related substrates and transporters. It is noteworthy that OCT1 and OCT2 showed opposite preferences for the enantiomers of the beta_2_-adrenergic agonist fenoterol. Regarding the high-affinity MATs, extensive studies have been performed on stereoselective transporter inhibition [32]. Many antidepressants acting as monoamine reuptake inhibitors are chiral, and transporter inhibition differs significantly between both enantiomers in several cases [33,34]. Consequently, chiral switching has been applied for several of the racemates assuming that the enantiopure drug may be more beneficial than the racemic mixture, as shown, for example, with escitalopram [35] or levomilnacipran [36]. Nevertheless, as monoamine transporter substrates have generally received less attention than their inhibitors, less is known about stereoselectivity in the transport by monoamine transporters. One of the few but intriguing examples of stereoselective transport is that of 3,4-methylendioxy-*N*-methylamphetamine. All MATs showed a highly selective transport of the *(R)*-enantiomer of this psychostimulant drug [37].

Recently, we have shown that the MAT substrate spectra are larger than previously known, and they efficiently transport numerous analogues of their name-giving substrates [38]. Additionally, there is a significant overlap in the substrate spectra of MATs and polyspecific OCT1, −2, and −3 [38]. Here, we stereospecifically characterize the uptake kinetics of 11 chiral monoamines, structurally related to epinephrine and norepinephrine.

By comparatively characterizing the transport of substances covering only a narrow chemical space, the observed transport activity can be related more easily to the structure of the substrates. Additionally, data on comparative analysis of typically high- and low-affinity organic cation transporters might be helpful to assess whether substrate affinity is related to stereoselectivity in drug membrane transport.

## 2. Materials and Methods

### 2.1. Test Compounds

Compounds were purchased from Neo-Biotech (Nanterre, France; NB-42-32196-1G, *(R)*-bisnorephedrine), Santa-Cruz Biotechnology (Darmstadt, Germany; sc-204086, milnacipran hydrochloride; sc357366-1G, l-norepinephrine), Sigma-Aldrich (Darmstadt, Germany; A72405-10G, bisnorephedrine; E4250-1G, l-epinephrine; E4642-5G, epinephrine hydrochloride; 209848-10G, halostachine; A0937-1G, norepinephrine bitartrate salt; N7127-100MG, normetanephrine; 113727-10G, norphenylephrine hydrochloride; O0250-1G, octopamine hydrochloride; P6126-5G, l-phenylephrine hydrochloride; SML0398-10MG, salsolinol hydrobromide; S0752-5G, synephrine) and Toronto Research Chemicals (Toronto, Canada; M258760, metanephrine; P320635, phenylephrine). The purity of all compounds used was at least 95% according to the respective manufacturers.

### 2.2. In-Vitro Transport Experiments

All transport experiments were carried out in HEK293 cells stably overexpressing human MATs or OCTs. NET, DAT, and SERT as well as OCT1 and OCT2 (wt and A270S variant) overexpressing cells were generated using the Flp-In system (Thermo Fisher Scientific, Darmstadt, Germany) as described previously [39,40,41]. OCT3 overexpressing cells were a kind gift from Drs Koepsell and Gorbulev (University of Würzburg, Würzburg, Germany). All cells were regularly cultivated in DMEM supplemented with 10% (*v*/*v*) FCS, penicillin (100 U/mL) and streptomycin (100 μg/mL)and kept in culture for no longer than 30 passages.

For the uptake experiments, 300,000 cells were plated in poly-d-lysine precoated 24-well plates 48 h ahead of the transport experiment. On the day of experiment, cells were initially washed once with 37 °C pre-warmed HBSS+ (Hanks buffered salt solution supplemented with 10 mM HEPES) and subsequently incubated with the substrates dissolved in pre-warmed HBSS+ for exactly two minutes. Incubation was stopped by adding ice-cold HBSS+ followed by two washing steps. After this, cells were lysed with 80% acetonitrile containing 10 ng/mL milnacipran as internal standard for high-performance liquid chromatography coupled to tandem mass spectrometry (HPLC-MS/MS) analysis. For every experiment, two additional wells per cell line were lysed using RIPA buffer for protein quantification. Total protein was quantified using the bicinchoninic acid assay [42]. This was later used for normalization of the uptake data.

### 2.3. Stereoselective Concentration Analyses

Intracellularly accumulated test substances were quantified by HPLC-MS/MS analysis using a Shimadzu Nexera HPLC system with a SIL-30AC autosampler, a CTO-20AC column oven, a LC-30AD pump, and a CBM-20A controller (Shimadzu, Kyoto, Japan). Stereoselective chromatography was carried out on a CHIRALPAK CBH HPLC column (100 × 3 mm inner dimension with 5 μM particle size; Sigma-Aldrich, Darmstadt, Germany) with a corresponding 10 × 3 mm guard column. Reversed phased chromatography was carried out using an ammonium acetate (NH_4_Ac) buffered aqueous mobile phase with isopropyl alcohol (IPA) as organic solvent. HPLC conditions are summarized for all analytes in Table S1. The order of elution of the enantiomers was obtained from available reference literature [43] or by applying enantiopure reference compounds to the chiral HPLC. For halostachine, metanephrine, normetanephrine, norphenylephrine, and salsolinol, the order of elution was not available. However, in every other case where the order of elution was available, the *(R)*-enantiomer eluted first. Given the structural similarity of the investigated substances and the fact that separation was performed on the same column with almost identical mobile phases, we assume that this order of elution for our compound set is always the *(R)*- before the *(S)*-enantiomer.

Detection was performed with an API 4000 tandem mass spectrometer (AB SCIEX, Darmstadt, Germany) operating in multiple-reaction-monitoring mode. The detection parameters are summarized in Table S2. Quantification was performed using the Analyst 1.6.2 software (AB SCIEX, Darmstadt, Germany) and quantified by comparison to standard curves with known concentrations.

### 2.4. Calculations

All uptake data were normalized to protein content to account for variation in the number of seeded sells. Transporter-mediated net uptake was calculated by subtracting uptake into empty vector-transfected control cells from uptake into transporter-transfected cells. Kinetic parameters were then determined after non-linear regression following the Michaelis–Menten equation (v = v_max_ × [S]/(K_m_ + [S])) using GraphPad Prism (Version 5.01 for Windows, GraphPad Software, La Jolla, CA, USA). V_max_ refers to the maximum transport velocity and K_m_ is defined as the substance concentration required to reach half of v_max_. The intrinsic clearance Cl_int_ was calculated as the quotient of v_max_ over K_m_.

## 3. Results

**Figure 2:**
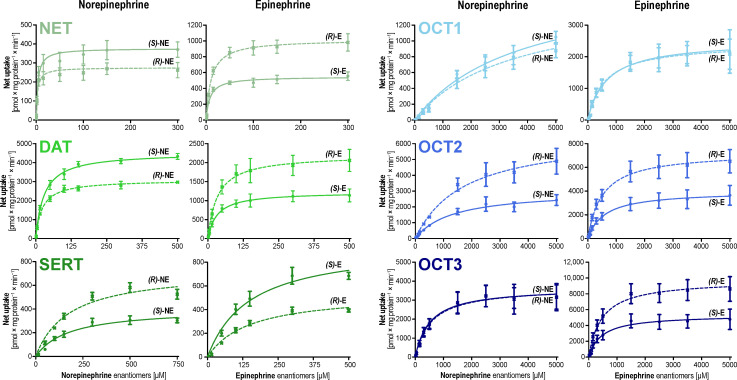
Stereoselective uptake of norepinephrine (NE) and epinephrine (E) by MATs and OCTs. Transporter-overexpressing HEK293 cells were incubated for two minutes with increasing concentrations of racemic norepinephrine and epinephrine. Data are provided as mean ± SEM of three independent experiments. Note that different scaling had to be applied in the graphs, particularly due to the substantially higher affinity of NET in comparison to the other transporters.

Initially, we investigated the stereoselective uptake of the monoamine neurotransmitters epinephrine and norepinephrine by MATs and OCTs (Figure 2).

NET showed the highest affinity for norepinephrine, while DAT exhibited the highest maximum transport capacities with both norepinephrine enantiomers (Table 1). SERT mediated uptake was characterized by a low affinity resulting in overall low intrinsic clearances of both epinephrine and norepinephrine enantiomers. With respect to stereoselectivity, interestingly, NET and DAT slightly preferred the uptake of *(S)*-norepinephrine over the pharmacologically much more active *(R)*-norepinephrine. This was switched for epinephrine, for which both transporters showed higher maximum transport activity for the active *(R)*-enantiomer with v_max_ ratios of about two-fold. However, the opposite was the case for SERT, which showed higher uptake of *(R)*-norepinephrine and *(S)*-epinephrine over their respective counterparts.

All three OCTs showed high-capacity transport for the enantiomers of both monoamines. Thus, they require a magnitude higher concentration to reach transporter saturation. The highest transport capacity was achieved by OCT3 for the active *(R)*-Epinephrine, which was 1.83-fold higher than the transport capacity for the corresponding *(S)*-enantiomer. In contrast, norepinephrine was not selectively transported by OCT3. Notably, OCT1 showed no selective uptake at all, while OCT2 was characterized by an approximately two-fold preference for the *(R)*-enantiomers of both epinephrine and norepinephrine.

**Figure 3:**
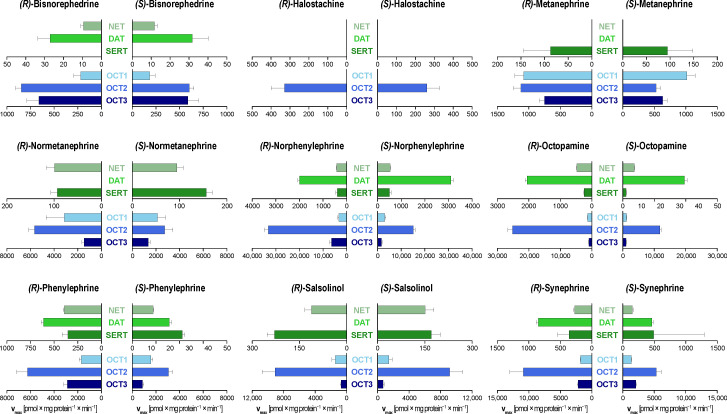
Maximum transport capacities, v_max_, of the investigated structural analogues of norepinephrine and epinephrine by MATs and OCTs. Data is presented as mean ± SEM of at least three independent experiments. Missing bars indicate no saturable net uptake by the respective transporters.

To characterize the stereoselectivity in the transport of phenylethylamines by both transporter families more thoroughly, we assessed the uptake of closely related chiral analogues of the monoamine neurotransmitters (Figure 3, Table S3). All net concentration-uptake curves are given in Figures S1–S6.

MATs accelerated the uptake of most but not all investigated compounds. The non-ring hydroxylated halostachine as well as the ring methoxylated analogues normetanephrine and metanephrine were no or only very weak MATs substrates. In contrast, the single ring-hydroxylated molecules norphenylephrine and octopamine and their *N*-methylated derivatives phenylephrine and synephrine were extraordinarily good substrates with intrinsic clearances similar to norepinephrine and epinephrine themselves, respectively (Table S3).

Concerning stereoselectivity, we observed similar patterns as seen with the transport of norepinephrine and epinephrine. For norphenylephrine and octopamine, NET and DAT showed only modest selectivity with a preference for the *(S)*-enantiomers. For the *N*-methylated analogues phenylephrine and synephrine, the uptake of the *(R)*-enantiomers was preferred. Again, SERT showed the opposite preference for norphenylephrine and phenylephrine. However, for octopamine, SERT had a 2.4-fold preference for the uptake of the *(R)*-enantiomer. Additionally, SERT also showed selective transport of the neurotoxic agent salsolinol.

**Figure 4:**
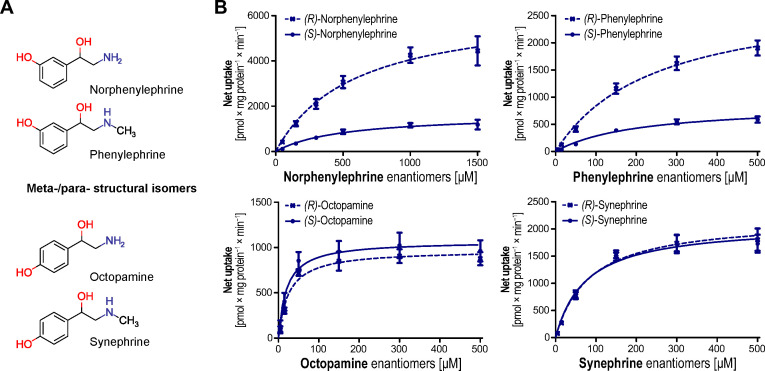
Chemical structures of single ring-hydroxylated phenylethylamine analogues (**A**). Stereoselective uptake analyses by OCT3 (**B**). Data is provided as mean ± SEM of three independent experiments.

OCT transport of investigated monoamines was generally characterized by a high capacity. All phenylethylamines were substrates of OCT2. This broad affinity of OCT2 for all phenylethylamine derivatives is particularly illustrated by the example of halostachine, which was not transported by any other transporter investigated here (Figure 3). OCT1 only showed stereoselective uptake for synephrine. In contrast, OCT2 transport was characterized by an approximately two-fold higher transport capacity for the *(R)*-enantiomers of all investigated phenylethylamines as compared to their *(S)*-enantiomers with the exception of salsolinol. OCT3 showed non-selective uptake for most substrates but showed the highest stereoselectivity observed in this study with norphenylephrine and phenylephrine (Figure 4). The *(R)*-enantiomers were transported with 3.9-fold and 3.3-fold higher transport capacities, respectively. Interestingly, their isomers with the phenolic hydroxyl group in para position, octopamine, and synephrine, showed no selective OCT3 uptake.

**Figure 5:**
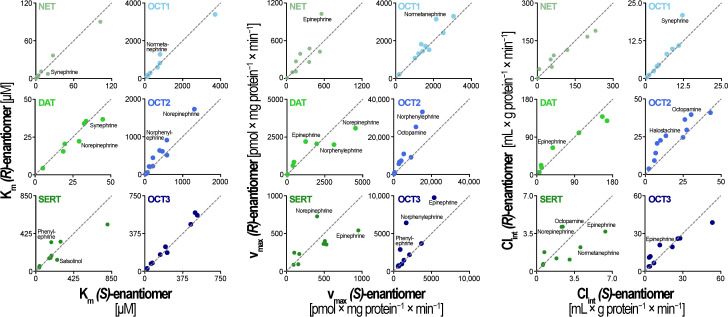
Analysis of stereoselectivity in the kinetic parameters K_m_, v_max_ and Cl_int_. Kinetic constants of the *(R)*-enantiomer uptake are shown on the ordinate whereas kinetic constants of the *(S)*-enantiomer are shown on the abscissa. The bisections are highlighted by the dashed lines, and stereoselectivity is indicated by how strongly each point deviates from these bisections.

A global comparison among all six transporters revealed that generally maximum transport capacities (v_max_) were more stereoselective than affinities (K_m_) (Figure 5). K_m_ values were less stereoselective, although OCT2 transported many enantiomers with different affinities. Interestingly, with several substrates, SERT showed higher stereoselectivity than NET and DAT, although SERT transport of the phenylethylamines was characterized by a lower affinity.

**Figure 6:**
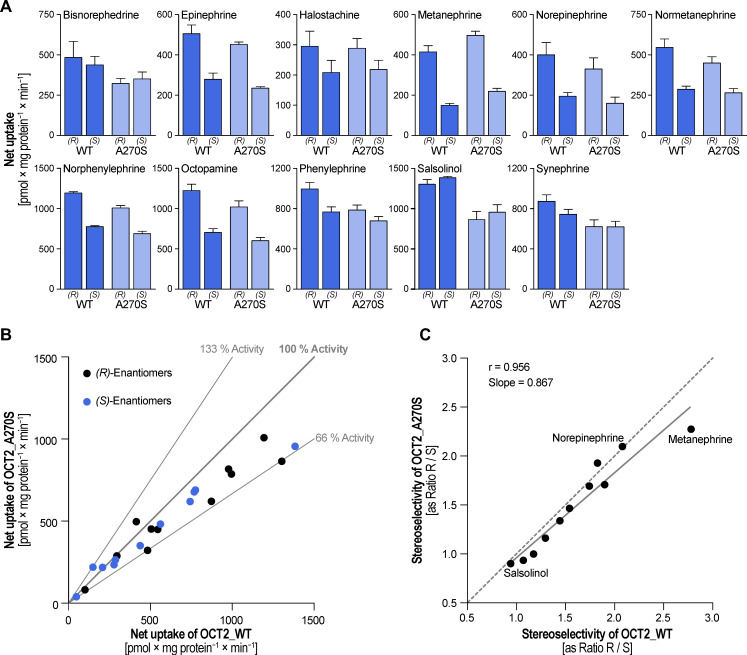
Influence of the A270S polymorphism on the stereoselectivity of monoamine transport by OCT2 (**A**). HEK293 cells stably overexpressing wild-type or mutant OCT2 were incubated with 100 μM racemic substance for two minutes. Data is shown as mean ± SEM of four independent experiments. Stereoselectivity and selectivity between both transporter variants are summarized in Table S4. Comparison of the net uptake by both transporter variants (**B**). The bisection indicates equal uptake, and the thin grey lines illustrate increased or decreased uptake, respectively. Correlation of stereoselectivity expressed as ratio of the uptake of the *(R)*- to the *(S)*-enantiomers (**C**). The bisection (dashed line) indicated equal stereoselectivity whereas the solid line illustrated the regression function.

Since OCT2 showed high uptake of all investigated compounds combined with a comparably strong stereoselectivity, we further tested whether the common A270S polymorphism affected uptake and selectivity (Figure 6A). In many human populations, this is the only non-synonymous OCT2 variant with functional impact and a reasonable population frequency.

The A270S variant affected transport activity with a slight increase in the case of metanephrine and a moderate reduction with all other substrates. Net uptake varied in comparison to the wild-type from 120% for *(R)*-metanephrine down to 66% for *(R)*-salsolinol (Figure 6B). However, stereoselectivity was highly similar (Figure 6C). Stereoselectivity of both variants showed a strong correlation (r = 0.96), although selectivity of the A270S was slightly lower as indicated by the slope of the regression line of 0.87.

## 4. Discussion

In this study, we comparatively characterized the transport of phenylethylamines by human monoamine transporters (MATs) and human organic cation transporters (OCTs) with a special focus on stereoselectivity. Stereoselectivity has been only sparingly investigated, although there is a high prevalence of chiral centers in numerous drug classes, and other pharmacological processes, such as metabolism and receptor binding, are known to be highly stereoselective. Stereoselectivity in the binding of several phenylethylamines to adrenoreceptors is summarized in Table 2. Generally, it is well established that *(R)*-(−)-epinephrine and *(R)*-(−)-norepinephrine are the pharmacologically active enantiomers binding with much higher affinity to the adrenergic alpha and beta receptors than their counterparts [44]. Additionally, binding to intracellular trace amine-associated receptors (TAARs) seems to also be highly stereoselective, as shown with the investigational TAAR1 agonist ulotaront [45].

Traditionally, MATs and OCTs appeared to be fundamentally different. MATs were characterized by narrow substrate spectra and were assigned to a clear physiological role as critical regulators of neuronal signal transmission [12]. In contrast to this, OCTs are polyspecific, have a broader tissue expression, and are involved in the pharmacokinetics of many drugs [17]. MAT uptake was described as high-affinity, low-capacity transport, while OCTs act as low-affinity, high-capacity transporters [49,50]. Recently, we studied the extended substrate spectra of MATs and characterized their substrate overlap with OCTs [38]. Especially, the substrate spectrum of OCT2 showed a high overlap with the substrate spectrum of the MATs, which is also highlighted by the present analyses (Figure 3, Table S3).

In general, our data confirmed the classification of MATs and OCTs as low- and high-capacity transporters for norepinephrine and epinephrine. However, the single ring-hydroxylated analogues of norepinephrine, norphenylephrine, and octopamine were taken up by NET with a similar or with an even higher intrinsic clearance as norepinephrine itself. Interestingly, there are exceptions from the simplified classification of MATs as high-affinity, low-capacity transporters and vice-versa for OCTs. DAT showed surprisingly high-capacity transport for several compounds, which was similar or even higher than that measured with OCT1 and OCT3, while DAT still maintains its high affinity (Figure S3). Additionally, OCT3 showed high-affinity transport of the octopamine enantiomers.

Among the OCTs, OCT2, in particular, showed extraordinarily high maximum transport capacities. All investigated substances were good substrates of OCT2 with mostly higher intrinsic clearances than those observed for OCT1 or OCT3. This is in line with previous observations regarding the specificity of OCTs, indicating OCT2 favored transport for small hydrophilic substances [38,51]. Moreover, this supports the role of neuronal OCT2 as an important player in the central clearance of monoamines [25,52,53]. With an allele frequency of around 10%, the tested A270S polymorphism is the most common non-synonymous polymorphism within the OCT2 coding region [54]. Here, we observed generally an only moderately reduced transport of monoamines by this variant compared to the wild type. Similar observations were also made for this variant for other substrates, including the psychostimulants ephedrine and dimethyltryptamine [39] and the nucleoside analogue lamivudine [55].

Stereoselectivity differed strongly between the investigated transporters. In particular, the three closely related OCTs showed substantial differences in their enantioselectivity. Whereas OCT1 transport was almost unselective at all, OCT2 showed a uniform stereoselectivity for all investigated phenylethylamines. In contrast, OCT3 showed non-selective uptake of several of the investigated compounds, but on the other hand, the highest stereoselectivity observed in this study was with norphenylephrine and phenylephrine. Interestingly, among the previously investigated adrenergic drugs and their stereoselective transport by OCTs, salbutamol showed a similarly high degree of stereoselectivity as well as a preference in the transport of its *(R)*-enantiomer by OCT3 [30]. Similar to norphenylephrine and phenylephrine, salbutamol also has an asymmetrically substituted phenol ring. Salbutamol has a larger methylhydroxyl group in the meta position. This may indicate that meta-substitutions of adrenergic drugs might determine OCT3 stereoselectivity, but more data on the stereoselective transport of structurally diverse analogues would be required to validate this hypothesis.

With MATs, we expected much higher stereoselectivity considering their higher affinity to the adrenergic drugs and the fact that evolution has “designed” these transporters, particularly for one of the enantiomers, and apparently the catecholamines are very old transmitters found in almost all species [56]. The lack of strong stereospecific uptake was also surprising since some inhibitors of these transporters act highly stereospecifically [57,58]. MAT stereoselectivity was not even higher as compared to the OCTs. Nevertheless, the series of here investigated substances revealed several interesting observations. First, NET and DAT generally showed the same stereoselectivity, which is not surprising given their high amino acid identity of approximately 67% and the similarity of their name-giving substrates. There, both transporters showed a minor enantiopreference for the *(S)*-enantiomer of norepinephrine, which was rather unexpected considering that NET is likely to be evolutionarily adapted to the transport of *(R)*-norepinephrine. However, this selectivity switches for epinephrine. Intriguingly, SERT, although characterized by an amino acid identity of approximately 50% towards NET and DAT, often showed the opposite stereoselectivity. This opposite stereospecific preference for closely related transporters highlights the difficulties of predicting transporter stereoselectivity. Nevertheless, SERT often showed higher stereoselectivity than NET and DAT, although SERT transport clearly showed lower affinity. This illustrates that stereoselectivity is not necessarily related to substrate affinity, at least in our data set.

The neurotransmitters norepinephrine and epinephrine, as well as most of their analogs investigated here, occur endogenously only as enantiopure *(R)*-enantiomers in human physiology, and thus, the physiological relevance of this data might appear to be limited. However, these data shed an interesting light on the apparently not highly specific molecular interactions between substrates and transporters. In addition, certain dietary supplements, used for instance as pre-workout in exercising, contain racemic synephrine [59], and some plant species seem to produce racemic synephrine as well [60]. For synephrine, as for most other phenylethylamines investigated in this study, the *(R)*-enantiomer is more potent in its pharmacodynamics than the *(S)*-enantiomer [48,61]. Stereoselective membrane transport, especially by peripherally expressed OCTs, might be highly relevant for the elimination of racemic synephrine from the body. Synephrine is rapidly removed from the bloodstream by hepatic uptake [62]. Thus, OCT1 is likely to be involved, and here we show a higher clearance for the active *(R)*-enantiomer. Another example of a substrate that is endogenously found as a racemate is salsolinol. Salsolinol is found as racemate in cocoa and cocoa-based foods [63], and racemic salsolinol is formed by non-enzymatic condensation of dopamine with acetaldehyde [64]. Salsolinol is neurotoxic [65], and cytotoxicity in human neuroblastoma cells was almost two-fold higher for its *(S)*-enantiomer [66]. Here, SERT showed stereoselective uptake of salsolinol with a preference for the less cytotoxic *(R)*-enantiomer.

Altogether, this study should contribute to the understanding of stereoselectivity in drug membrane transport. Our data show that stereoselectivity is highly transporter-to-substrate specific, and closely related transporters differ in their selectivity. Moreover, stereoselectivity is highly substance-specific, and single structural alterations might lead to a completely different selectivity. All this highlights that more studies on stereoselectivity in drug membrane transport are required to fully evaluate its potential impact on the transport of chiral drugs and endogenous substances. In particular, the stereoselectivity found with OCT2 and (with some substances) with OCT3 may be relevant in neurotransmission considering the wide expression of these two transporters in the human brain.

## Figures and Tables

**Table 1:** Transport kinetic parameters (K_m_, apparent affinity; v_max_, maximum transport capacity; Cl_int_, intrinsic clearance) of norepinephrine and epinephrine enantiomers by MATs and OCTs.

Transporter	Substrate	K_m_ ± SEM [µM]	V_max_ ± SEM [pmol × mg Protein^−1^ × min^−1^]	Cl_int_ ± SEM [mL × g Protein^−1^ × min^−1^]	Stereoselectivity		
					K_m_	V_max_	Cl_Int_
NET	*(R)*-Norepinephrine	2.31 ± 0.84	262 ± 15	113.3 ± 47.6	1.32-fold for *(S)*	1.38-fold for (*S*) **	1.05-fold for *(S)*
	*(S)*-Norepinephrine	3.05 ± 1.01	362 ± 20	118.7 ± 46.0			
	*(R)*-Epinephrine	11.2 ± 2.2	1027 ± 37	91.7 ± 21.3	1.31-fold for *(R)*	1.84-fold for *(R)* ***	1.41-fold for *(R)*
	*(S)*-Epinephrine	8.52 ± 1.91	557 ± 22	65.4 ± 17.3			
DAT	*(R)*-Norepinephrine	22.2 ± 2.6	3085 ± 81	138.8 ± 19.7	1.31-fold for *(S)*	1.47-fold for *(S)* ***	1.12-fold for *(S)*
	*(S)*-Norepinephrine	29.1 ± 3.2	4524 ± 122	155.5 ± 21.4			
	*(R)*-Epinephrine	34.0 ± 11.6	2201 ± 173	64.7 ± 26.7	1.05-fold for *(R)*	1.80-fold for *(R)* **	1.71-fold for *(R)*
	*(S)*-Epinephrine	32.3 ± 10.3	1226 ± 90	37.9 ± 14.8			
SERT	*(R)*-Norepinephrine	176.5 ± 41.7	727 ± 60	4.12 ± 1.31	1.01-fold for *(R)*	1.79-fold for *(R)* **	1.78-fold for *(R)*
	*(S)*-Norepinephrine	175.4 ± 52.23	405 ± 42	2.31 ± 0.93			
	*(R)*-Epinephrine	147.1 ± 27.6	542 ± 41	3.68 ± 0.97	1.02-fold for *(S)*	1.76-fold for *(S)* **	1.73-fold for *(S)*
	*(S)*-Epinephrine	149.6 ± 35.6	951 ± 91	6.35 ± 2.12			
OCT1	*(R)*-Norepinephrine	3394 ± 1417	1524 ± 330	0.45 ± 0.28	1.09-fold for *(S)*	1.16-fold for *(S)*	1.06-fold for *(S)*
	*(S)*-Norepinephrine	3710 ± 1011	1766 ± 258	0.48 ± 0.20			
	*(R)*-Epinephrine	596.1 ± 154.6	2419 ± 189	4.06 ± 1.37	1.13-fold for *(S)*	1.04-fold for *(S)*	1.08-fold for *(R)*
	*(S)*-Epinephrine	670.7 ± 230.3	2514 ± 266	3.75 ± 1.68			
OCT2	*(R)*-Norepinephrine	1731 ± 417.5	6603 ± 664	3.81 ± 1.30	1.32-fold for *(R)*	2.17-fold for *(R)* **	1.64-fold for *(R)*
	*(S)*-Norepinephrine	1309 ± 290.9	3042 ± 258	2.32 ± 0.71			
	*(R)*-Epinephrine	513.1 ± 110.4	7277 ± 483	14.2 ± 4.0	1.14-fold for *(S)*	1.82-fold for *(R)* **	2.08-fold for *(R)*
	*(S)*-Epinephrine	584.6 ± 167.4	3993 ± 360	6.83 ± 2.57			
OCT3	*(R)*-Norepinephrine	557.1 ± 200.0	3683 ± 360	6.61 ± 3.02	1.07-fold for *(R)*	1.01-fold for *(R)*	1.07-fold for *(S)*
	*(S)*-Norepinephrine	519.2 ± 158.5	3662 ± 299	7.05 ± 2.73			
	*(R)*-Epinephrine	466.5 ± 116.0	9742 ± 698	20.9 ± 6.7	1.03-fold for *(R)*	1.83-fold for *(R)* **	1.78-fold for *(S)*
	*(S)*-Epinephrine	454.9 ± 149.0	5336 ± 502	11.7 ± 4.9			

SEM, standard error of the mean; asterisks indicate statistical significance of the differences between the two enantiomers (Student’s *t*-test; ** *p* < 0.01, *** *p* < 0.001).

**Table 2:** Stereoselectivity in adrenoreceptor binding by phenylethylamines investigated here.

Substance	Enantiomeric Activity Ratio (R/S) in Receptor Binding			References
	Alpha-1	Alpha-2	Beta	
Epinephrine	49		125	[46,47]
Norepinephrine	33			[48]
Norphenylephrine	8	3		[48]
Octopamine	5	3		[48]
Phenylephrine	420	45		[48]
Synephrine	75			[48]

## Data Availability

The raw data supporting the conclusions of this article will be made available by the authors, without undue reservation.

## Supplementary Materials

The following supporting information can be downloaded at: https://www.mdpi.com/article/10.3390/biom12101507/s1, Figure S1: Net uptake curves of the norepinephrine transporter (NET), Figure S2: Net uptake curves of the dopamine transporter (DAT), Figure S3: Net uptake curves of the serotonin transporter (SERT), Figure S4: Net uptake curves of the organic cation transporter 1 (OCT1), Figure S5: Net uptake curves of the organic cation transporter 2 (OCT2), Figure S6: Net uptake curves of the organic cation transporter 3 (OCT3); Table S1: HPLC conditions for chiral separation of investigated substances, Table S2: Mass spectrometry detection parameters, Table S3: Kinetic parameters for the stereoselective transport of chiral phenylethylamines by MATs and OCTs, Table S4: Stereoselectivity and transporter selectivity of investigated phenylethylamines for OCT2 and its A270S variant.

## References

[1] Libersat F., Pflueger H.-J. (2004). Monoamines and the Orchestration of Behavior. BioScience.

[2] Gainetdinov R.R., Hoener M.C., Berry M.D. (2018). Trace Amines and Their Receptors. Pharmacol. Rev..

[3] Boulton A.A. (1974). Letter: Amines and theories in psychiatry. Lancet.

[4] Small K.M., McGraw D.W., Liggett S.B. (2003). Pharmacology and physiology of human adrenergic receptor polymorphisms. Annu. Rev. Pharmacol. Toxicol..

[5] Hernandez M.A., Rathinavelu A. (2017). Basic Pharmacology: Understanding Drug Actions and Reactions.

[6] Danielson T.J., Boulton A.A., Robertson H.A. (1977). m-Octopamine, p-octopamine and phenylethanolamine in rat brain: A sensitive, specific assay and the effects of some drugs. J. Neurochem..

[7] Boulton A.A., Wu P.H. (1972). Biosynthesis of cerebral phenolic amines. I. In vivo formation of p-tyramine, octopamine, and synephrine. Can. J. Biochem..

[8] Boulton A.A., Wu P.H. (1973). Biosynthesis of cerebral phenolic amines. II. In vivo regional formation of p-tyramine and octopamine from tyrosine and dopamine. Can. J. Biochem..

[9] Lamouroux A., Vigny A., Faucon Biguet N., Darmon M.C., Franck R., Henry J.P., Mallet J. (1987). The primary structure of human dopamine-beta-hydroxylase: Insights into the relationship between the soluble and the membrane-bound forms of the enzyme. EMBO J..

[10] Naoi M., Maruyama W., Dostert P., Kohda K., Kaiya T. (1996). A novel enzyme enantio-selectively synthesizes (R)-salsolinol, a precursor of a dopaminergic neurotoxin, N-methyl(R)salsolinol. Neurosci. Lett..

[11] Mack F., Bönisch H. (1979). Dissociation constants and lipophilicity of catecholamines and related compounds. Naunyn-Schmiedeberg’s Arch. Pharmacol..

[12] Kristensen A.S., Andersen J., Jørgensen T.N., Sørensen L., Eriksen J., Loland C.J., Strømgaard K., Gether U. (2011). SLC6 neurotransmitter transporters: Structure, function, and regulation. Pharmacol. Rev..

[13] Daws L.C. (2009). Unfaithful neurotransmitter transporters: Focus on serotonin uptake and implications for antidepressant efficacy. Pharmacol. Ther..

[14] Gu H., Wall S.C., Rudnick G. (1994). Stable expression of biogenic amine transporters reveals differences in inhibitor sensitivity, kinetics, and ion dependence. J. Biol. Chem..

[15] Nirenberg M.J., Vaughan R.A., Uhl G.R., Kuhar M.J., Pickel V.M. (1996). The dopamine transporter is localized to dendritic and axonal plasma membranes of nigrostriatal dopaminergic neurons. J. Neurosci. Off. J. Soc. Neurosci..

[16] Duan H., Wang J. (2010). Selective transport of monoamine neurotransmitters by human plasma membrane monoamine transporter and organic cation transporter 3. J. Pharmacol. Exp. Ther..

[17] Koepsell H. (2020). Organic Cation Transporters in Health and Disease. Pharmacol. Rev..

[18] Seitz T., Stalmann R., Dalila N., Chen J., Pojar S., Dos Santos Pereira J.N., Krätzner R., Brockmöller J., Tzvetkov M.V. (2015). Global genetic analyses reveal strong inter-ethnic variability in the loss of activity of the organic cation transporter OCT1. Genome Med..

[19] Hilgendorf C., Ahlin G., Seithel A., Artursson P., Ungell A.L., Karlsson J. (2007). Expression of thirty-six drug transporter genes in human intestine, liver, kidney, and organotypic cell lines. Drug Metab. Dispos. Biol. Fate Chem..

[20] Wu X., Kekuda R., Huang W., Fei Y.J., Leibach F.H., Chen J., Conway S.J., Ganapathy V. (1998). Identity of the organic cation transporter OCT3 as the extraneuronal monoamine transporter (uptake2) and evidence for the expression of the transporter in the brain. J. Biol. Chem..

[21] Busch A.E., Karbach U., Miska D., Gorboulev V., Akhoundova A., Volk C., Arndt P., Ulzheimer J.C., Sonders M.S., Baumann C. (1998). Human neurons express the polyspecific cation transporter hOCT2, which translocates monoamine neurotransmitters, amantadine, and memantine. Mol. Pharmacol..

[22] Chen E.C., Matsson P., Azimi M., Zhou X., Handin N., Yee S.W., Artursson P., Giacomini K.M. (2022). High Throughput Screening of a Prescription Drug Library for Inhibitors of Organic Cation Transporter 3, OCT3. Pharm. Res..

[23] Bönisch H. (1980). Extraneuronal transport of catecholamines. Pharmacology.

[24] Gasser P.J. (2021). Organic Cation Transporters in Brain Catecholamine Homeostasis.

[25] Bacq A., Balasse L., Biala G., Guiard B., Gardier A.M., Schinkel A., Louis F., Vialou V., Martres M.P., Chevarin C. (2012). Organic cation transporter 2 controls brain norepinephrine and serotonin clearance and antidepressant response. Mol. Psychiatry.

[26] Vialou V., Balasse L., Callebert J., Launay J.M., Giros B., Gautron S. (2008). Altered aminergic neurotransmission in the brain of organic cation transporter 3-deficient mice. J. Neurochem..

[27] Inazu M., Takeda H., Matsumiya T. (2003). Expression and functional characterization of the extraneuronal monoamine transporter in normal human astrocytes. J. Neurochem..

[28] Zhou Q., Yu L.-S., Zeng S. (2014). Stereoselectivity of chiral drug transport: A focus on enantiomer–transporter interaction. Drug Metab. Rev..

[29] Bi Y.A., Lin J., Mathialagan S., Tylaska L., Callegari E., Rodrigues A.D., Varma M.V.S. (2018). Role of Hepatic Organic Anion Transporter 2 in the Pharmacokinetics of R- and S-Warfarin: In Vitro Studies and Mechanistic Evaluation. Mol. Pharm..

[30] Jensen O., Rafehi M., Tzvetkov M.V., Brockmöller J. (2020). Stereoselective cell uptake of adrenergic agonists and antagonists by organic cation transporters. Biochem. Pharmacol..

[31] Gebauer L., Arul Murugan N., Jensen O., Brockmöller J., Rafehi M. (2021). Molecular basis for stereoselective transport of fenoterol by the organic cation transporters 1 and 2. Biochem. Pharmacol..

[32] Quinn D. (2008). Does chirality matter? pharmacodynamics of enantiomers of methylphenidate in patients with attention-deficit/hyperactivity disorder. J. Clin. Psychopharmacol..

[33] Nishimura M., Sato K. (1999). Ketamine stereoselectively inhibits rat dopamine transporter. Neurosci. Lett..

[34] Niello M., Cintulová D., Raithmayr P., Holy M., Jäntsch K., Colas C., Ecker G.F., Sitte H.H., Mihovilovic M.D. (2021). Effects of Hydroxylated Mephedrone Metabolites on Monoamine Transporter Activity in vitro. Front. Pharmacol..

[35] Lepola U., Wade A., Andersen H.F. (2004). Do equivalent doses of escitalopram and citalopram have similar efficacy? A pooled analysis of two positive placebo-controlled studies in major depressive disorder. Int. Clin. Psychopharmacol..

[36] Auclair A.L., Martel J.C., Assié M.B., Bardin L., Heusler P., Cussac D., Marien M., Newman-Tancredi A., O’Connor J.A., Depoortère R. (2013). Levomilnacipran (F2695), a norepinephrine-preferring SNRI: Profile in vitro and in models of depression and anxiety. Neuropharmacology.

[37] Verrico C.D., Miller G.M., Madras B.K. (2007). MDMA (Ecstasy) and human dopamine, norepinephrine, and serotonin transporters: Implications for MDMA-induced neurotoxicity and treatment. Psychopharmacology.

[38] Gebauer L., Jensen O., Neif M., Brockmöller J., Dücker C. (2021). Overlap and Specificity in the Substrate Spectra of Human Monoamine Transporters and Organic Cation Transporters 1, 2 and 3. Int. J. Mol. Sci..

[39] Jensen O., Rafehi M., Gebauer L., Brockmöller J. (2021). Cellular Uptake of Psychostimulants—Are High- and Low-Affinity Organic Cation Transporters Drug Traffickers?. Front. Pharmacol..

[40] Dos Santos Pereira J.N., Tadjerpisheh S., Abu Abed M., Saadatmand A.R., Weksler B., Romero I.A., Couraud P.O., Brockmöller J., Tzvetkov M.V. (2014). The poorly membrane permeable antipsychotic drugs amisulpride and sulpiride are substrates of the organic cation transporters from the SLC22 family. AAPS J..

[41] Saadatmand A.R., Tadjerpisheh S., Brockmöller J., Tzvetkov M.V. (2012). The prototypic pharmacogenetic drug debrisoquine is a substrate of the genetically polymorphic organic cation transporter OCT1. Biochem. Pharmacol..

[42] Smith P.K., Krohn R.I., Hermanson G.T., Mallia A.K., Gartner F.H., Provenzano M.D., Fujimoto E.K., Goeke N.M., Olson B.J., Klenk D.C. (1985). Measurement of protein using bicinchoninic acid. Anal. Biochem..

[43] Pellati F., Benvenuti S., Melegari M. (2005). Enantioselective LC analysis of synephrine in natural products on a protein-based chiral stationary phase. J. Pharm. Biomed. Anal..

[44] Wieland K., Zuurmond H.M., Krasel C., Ijzerman A.P., Lohse M.J. (1996). Involvement of Asn-293 in stereospecific agonist recognition and in activation of the beta 2-adrenergic receptor. Proc. Natl. Acad. Sci. USA.

[45] Heffernan M.L.R., Herman L.W. (2022). Ulotaront: A TAAR1 Agonist for the Treatment of Schizophrenia. ACS Med. Chem. Lett..

[46] Rice P.J., Miller D.D., Patil P.N. (1989). Epinephrine enantiomers: Affinity, efficacy and potency relationships in rat smooth muscle tissues. J. Pharmacol. Exp. Ther..

[47] Brown E.M., Fedak S.A., Woodard C.J., Aurbach G.D. (1976). Beta-Adrenergic receptor interactions. Direct comparison of receptor interaction and biological activity. J. Biol. Chem..

[48] Brown C.M., McGrath J.C., Midgley J.M., Muir A.G., O’Brien J.W., Thonoor C.M., Williams C.M., Wilson V.G. (1988). Activities of octopamine and synephrine stereoisomers on alpha-adrenoceptors. Br. J. Pharmacol..

[49] Eisenhofer G. (2001). The role of neuronal and extraneuronal plasma membrane transporters in the inactivation of peripheral catecholamines. Pharmacol. Ther..

[50] Iversen L.L. (1965). The uptake of catechol amines at high perfusion concentrations in the rat isolated heart: A novel catechol amine uptake process. Br. J. Pharmacol. Chemother..

[51] Hendrickx R., Johansson J.G., Lohmann C., Jenvert R.-M., Blomgren A., Börjesson L., Gustavsson L. (2013). Identification of Novel Substrates and Structure–Activity Relationship of Cellular Uptake Mediated by Human Organic Cation Transporters 1 and 2. J. Med. Chem..

[52] Berry M.D., Hart S., Pryor A.R., Hunter S., Gardiner D. (2016). Pharmacological characterization of a high-affinity p-tyramine transporter in rat brain synaptosomes. Sci. Rep..

[53] Taubert D., Grimberg G., Stenzel W., Schömig E. (2007). Identification of the endogenous key substrates of the human organic cation transporter OCT2 and their implication in function of dopaminergic neurons. PLoS ONE.

[54] Leabman M.K., Huang C.C., Kawamoto M., Johns S.J., Stryke D., Ferrin T.E., DeYoung J., Taylor T., Clark A.G., Herskowitz I. (2002). Polymorphisms in a human kidney xenobiotic transporter, OCT2, exhibit altered function. Pharmacogenetics.

[55] Choi M.K., Song I.S. (2012). Genetic variants of organic cation transporter 1 (OCT1) and OCT2 significantly reduce lamivudine uptake. Biopharm. Drug Dispos..

[56] Gallo V.P., Accordi F., Chimenti C., Civinini A., Crivellato E. (2016). Catecholaminergic System of Invertebrates: Comparative and Evolutionary Aspects in Comparison With the Octopaminergic System. Int. Rev. Cell Mol. Biol..

[57] Lane R.M., Baker G.B. (1999). Chirality and drugs used in psychiatry: Nice to know or need to know?. Cell. Mol. Neurobiol..

[58] Sánchez C., Bergqvist P.B.F., Brennum L.T., Gupta S., Hogg S., Larsen A., Wiborg O. (2003). Escitalopram, the S-(+)-enantiomer of citalopram, is a selective serotonin reuptake inhibitor with potent effects in animal models predictive of antidepressant and anxiolytic activities. Psychopharmacology.

[59] Koh A.H.W., Chess-Williams R., Lohning A.E. (2021). Racemic synephrine found in Citrus aurantium-listing pre-workout supplements suggests a non-plant-based origin. Drug Test. Anal..

[60] Ranieri R.L., McLaughlin J.L. (1976). Cactus alkaloids. XXVIII. .beta.-Phenethylamine and tetrahydroisoquinoline alkaloids from the Mexican cactus Dolichothele longimamma. J. Org. Chem..

[61] Jordan R., Midgley J.M., Thonoor C.M., Williams C.M. (1987). Beta-adrenergic activities of octopamine and synephrine stereoisomers on guinea-pig atria and trachea. J. Pharm. Pharmacol..

[62] da Silva-Pereira J.F., Bubna G.A., Gonçalves Gde A., Bracht F., Peralta R.M., Bracht A. (2016). Fast hepatic biotransformation of p-synephrine and p-octopamine and implications for their oral intake. Food Funct..

[63] Melzig M.F., Putscher I., Henklein P., Haber H. (2000). In vitro pharmacological activity of the tetrahydroisoquinoline salsolinol present in products from *Theobroma cacao* L. like cocoa and chocolate. J. Ethnopharmacol..

[64] Lee J., Ramchandani V.A., Hamazaki K., Engleman E.A., McBride W.J., Li T.-K., Kim H.-Y. (2010). A critical evaluation of influence of ethanol and diet on salsolinol enantiomers in humans and rats. Alcohol. Clin. Exp. Res..

[65] Kurnik-Łucka M., Panula P., Bugajski A., Gil K. (2018). Salsolinol: An Unintelligible and Double-Faced Molecule—Lessons Learned from In Vivo and In Vitro Experiments. Neurotox. Res..

[66] Takahashi T., Maruyama W., Deng Y., Dostert P., Nakahara D., Niwa T., Ohta S., Naoi M. (1997). Cytotoxicity of endogenous isoquinolines to human dopaminergic neuroblastoma SH-SY5Y cells. J. Neural Transm..

