# Supplementary material for: Stereoselectivity in the Membrane Transport of Phenylethylamine Derivatives by Human Monoamine Transporters and Organic Cation Transporters 1, 2, and 3
Source: Biomolecules. 2022 Oct 18;12(10):1507. doi: 10.3390/biom12101507 (PMC9599461; doi:10.3390/biom12101507)
Supplement: Supplementary file 1 [file biomolecules-12-01507-s001.zip › biomolecules-1948923-supplementary.pdf]

Supporting information

Stereoselectivity in the Membrane Transport of Phenylethylamine Derivatives by Human Monoamine Transporters and Organic Cation Transporters 1, 2, and 3

Lukas Gebauer*, Muhammad Rafehi and Jürgen Brockmöller

Institute of Clinical Pharmacology, University Medical Center Göttingen, D-37075 Göttingen, Germany

E-Mail: lukas.gebauer@med.uni-goettingen.de

Table of contents

Figure S1: Net uptake curves of the norepinephrine transporter (NET)

Figure S2: Net uptake curves of the dopamine transporter (DAT)

Figure S3: Net uptake curves of the serotonin transporter (SERT)

Figure S4: Net uptake curves of the organic cation transporter 1 (OCT1)

Figure S5: Net uptake curves of the organic cation transporter 2 (OCT2)

Figure S6: Net uptake curves of the organic cation transporter 3 (OCT3)

Table S1: HPLC conditions for chiral separation of investigated substances

Table S2: Mass spectrometry detection parameters

Table S3: Kinetic parameters for the stereoselective transport of chiral phenylethylamines by MATs and OCTs

Table S4: Stereoselectivity and transporter selectivity of investigated phenylethylamines for OCT2 and its A270S variant

Norepinephrine Transporter (NET)/*SLC6A2*

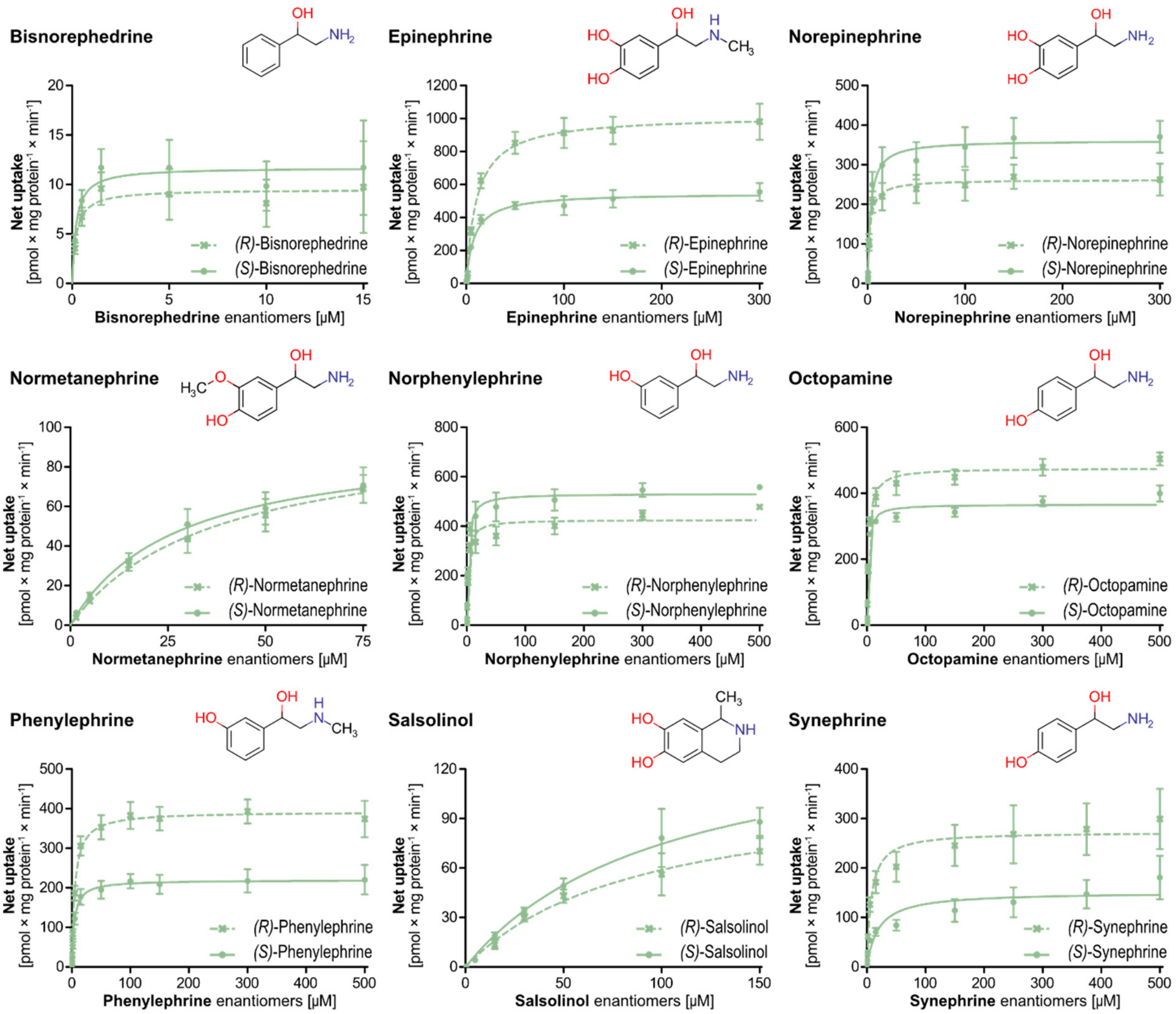

Figure S1 Net uptake curves of the norepinephrine transporter (NET) for investigated chiral phenylethylamines (in alphabetical order). Data is presented as mean ± SEM of at least three independent experiments. Not transported substances (no net uptake) are not shown.

Dopamine Transporter (DAT)/*SLC6A3*

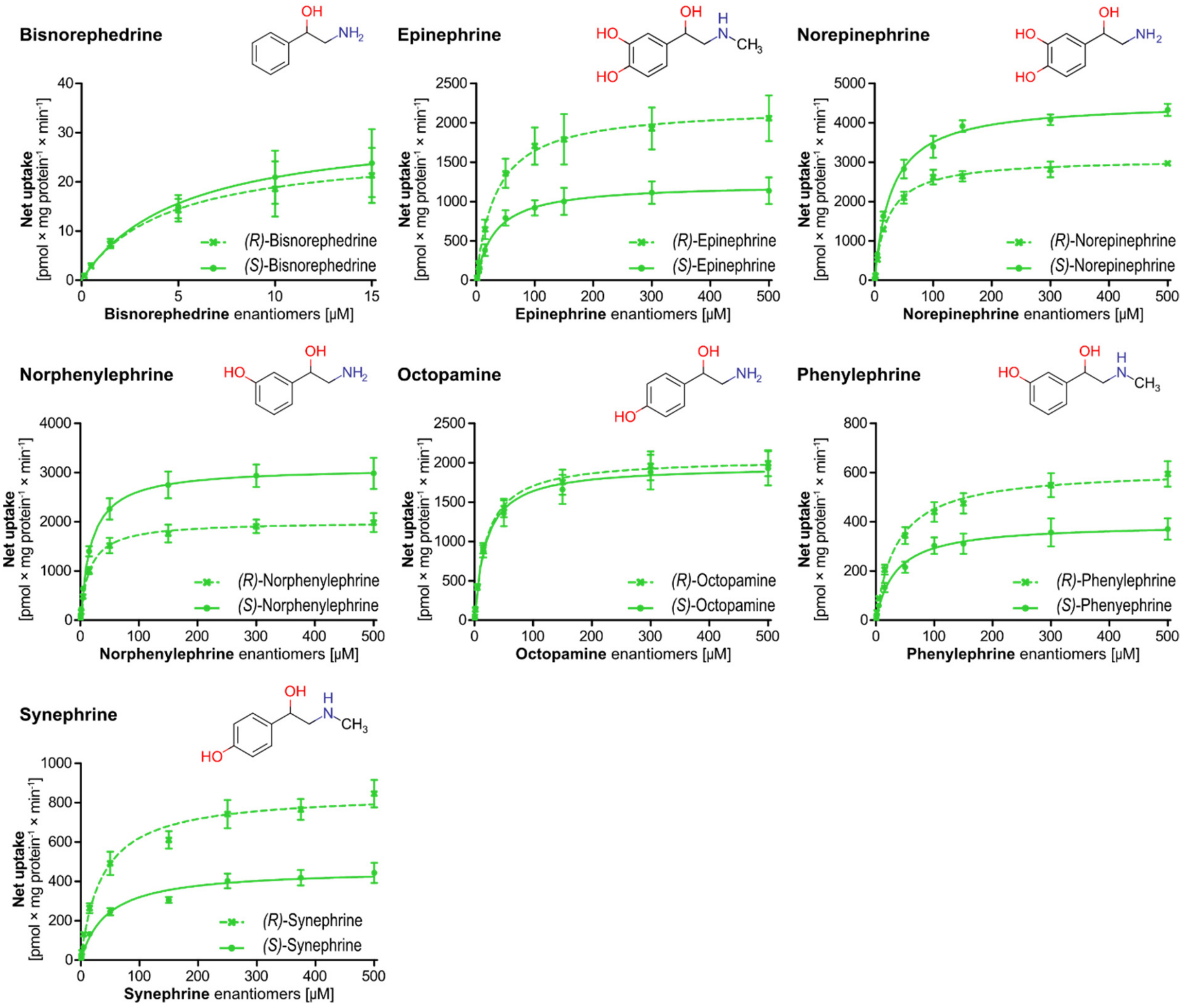

Figure S2 Net uptake curves of the dopamine transporter (DAT) for investigated chiral phenylethylamines (in alphabetical order). Data is presented as mean ± SEM of at least three independent experiments. Not transported substances (no net uptake) are not shown.

Serotonin Transporter (SERT)/*SLC6A4*

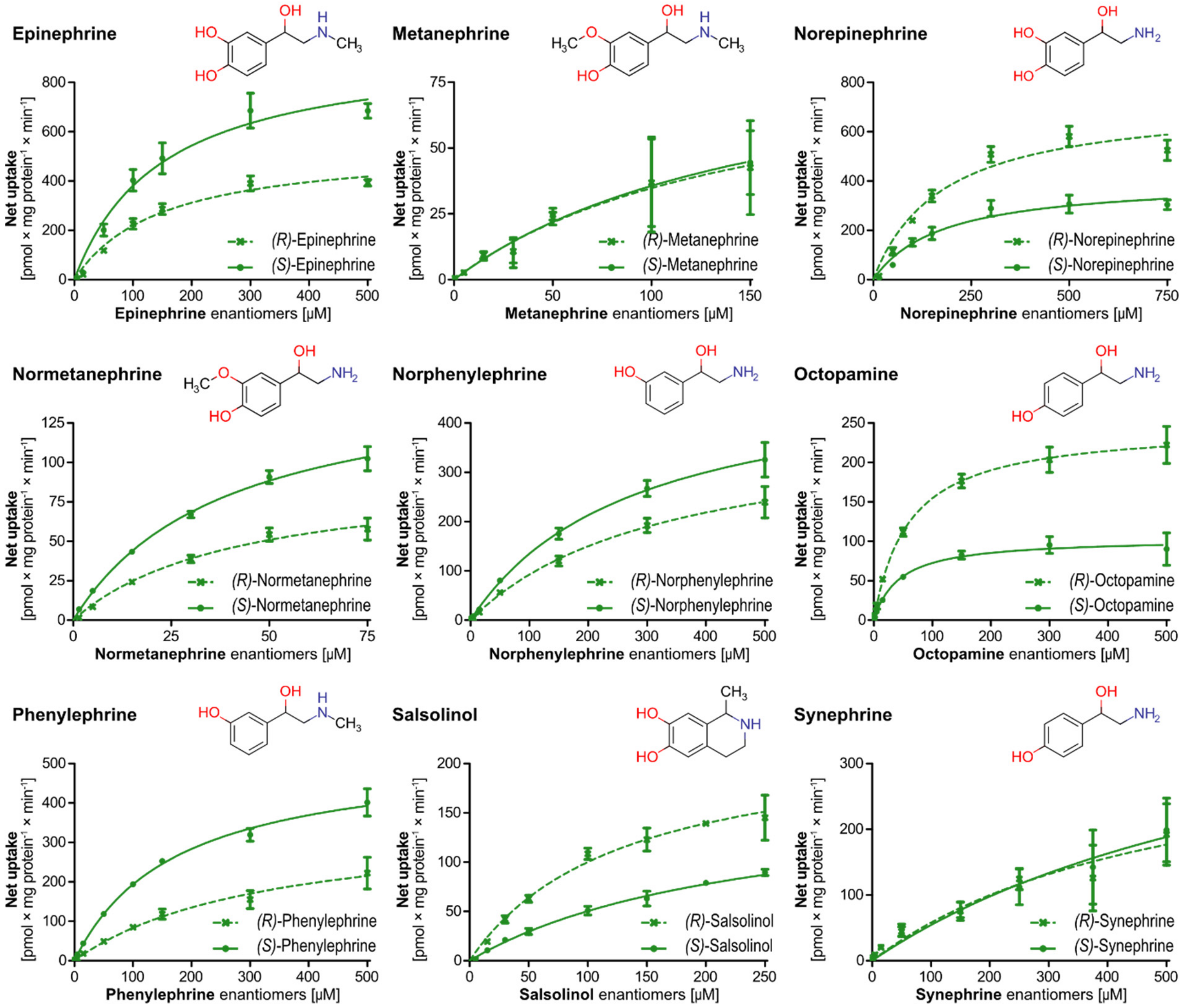

Figure S3 Net uptake curves of the serotonin transporter (SERT) for investigated chiral phenylethylamines (in alphabetical order). Data is presented as mean ± SEM of at least three independent experiments. Not transported substances (no net uptake) are not shown.

Organic cation transporter 1 (OCT1)/*SLC22A1*

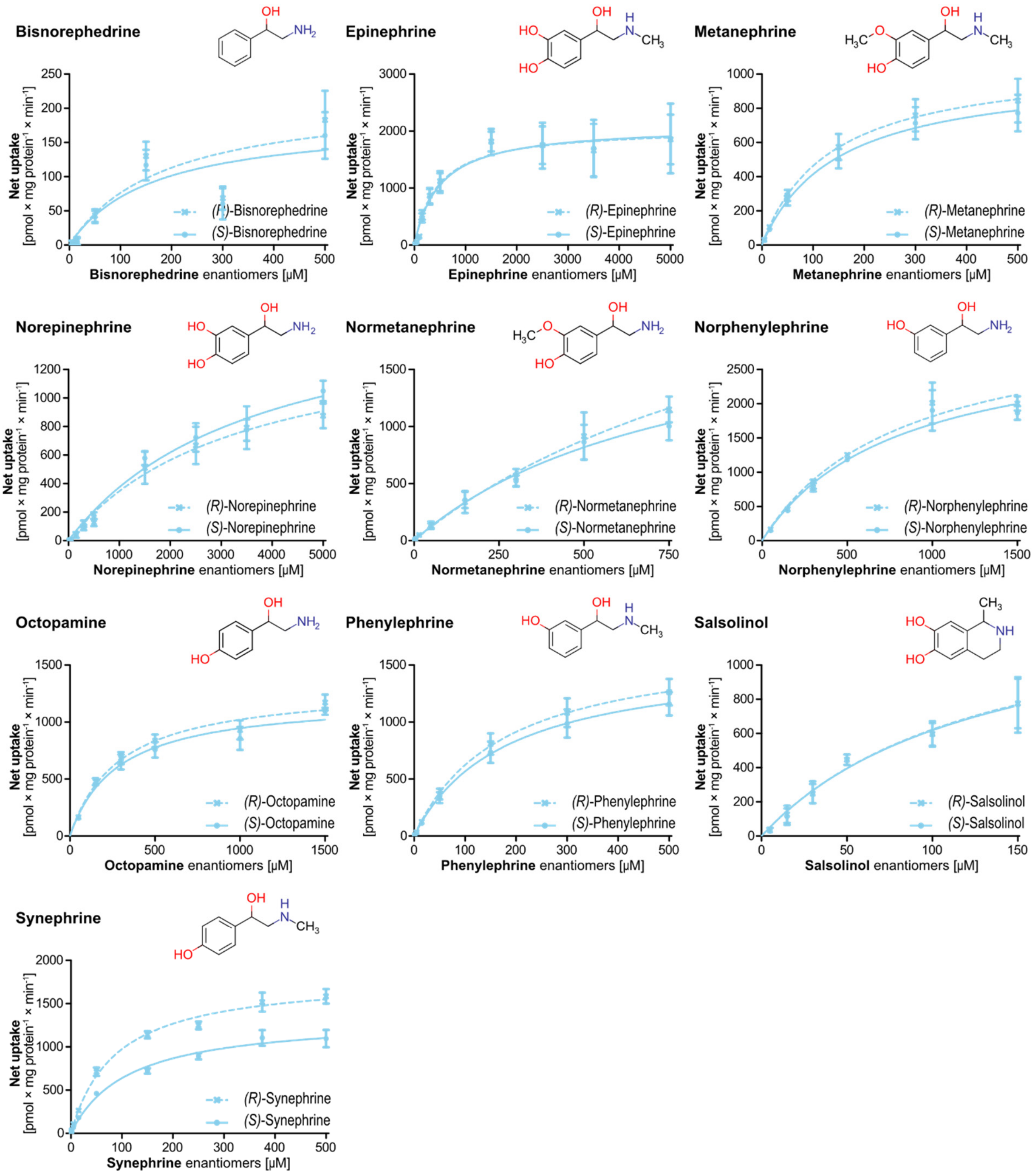

Figure S4 Net uptake curves of the organic cation transporter 1 (OCT1) for investigated chiral phenylethylamines (in alphabetical order). Data is presented as mean ± SEM of at least three independent experiments. Not transported substances (no net uptake) are not shown.

Organic cation transporter 2 (OCT2)/*SLC22A2*

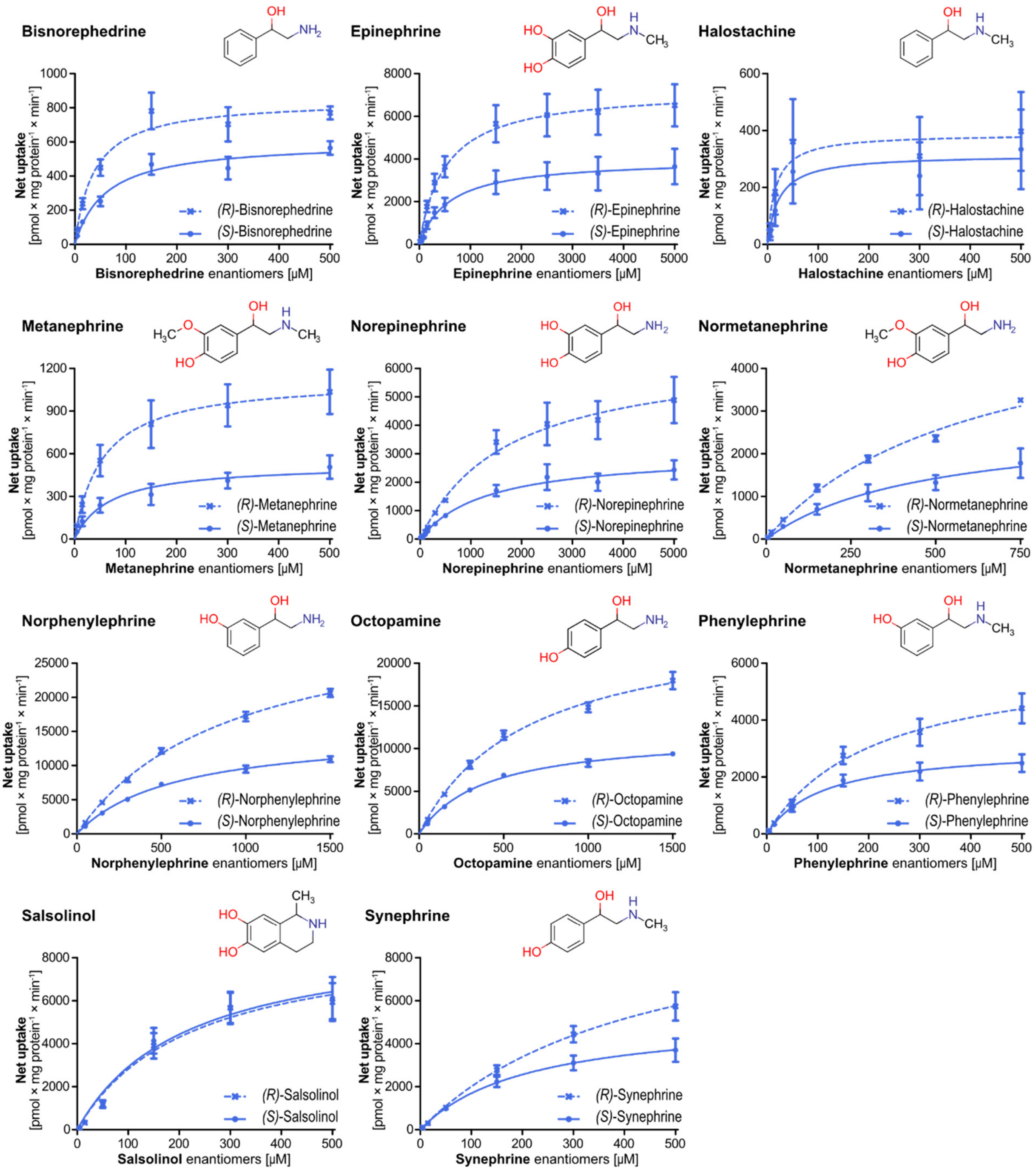

Figure S5 Net uptake curves of the organic cation transporter 2 (OCT2) for investigated chiral phenylethylamines (in alphabetical order). Data is presented as mean ± SEM of at least three independent experiments. Not transported substances (no net uptake) are not shown.

Organic cation transporter 3 (OCT1)/*SLC22A3*

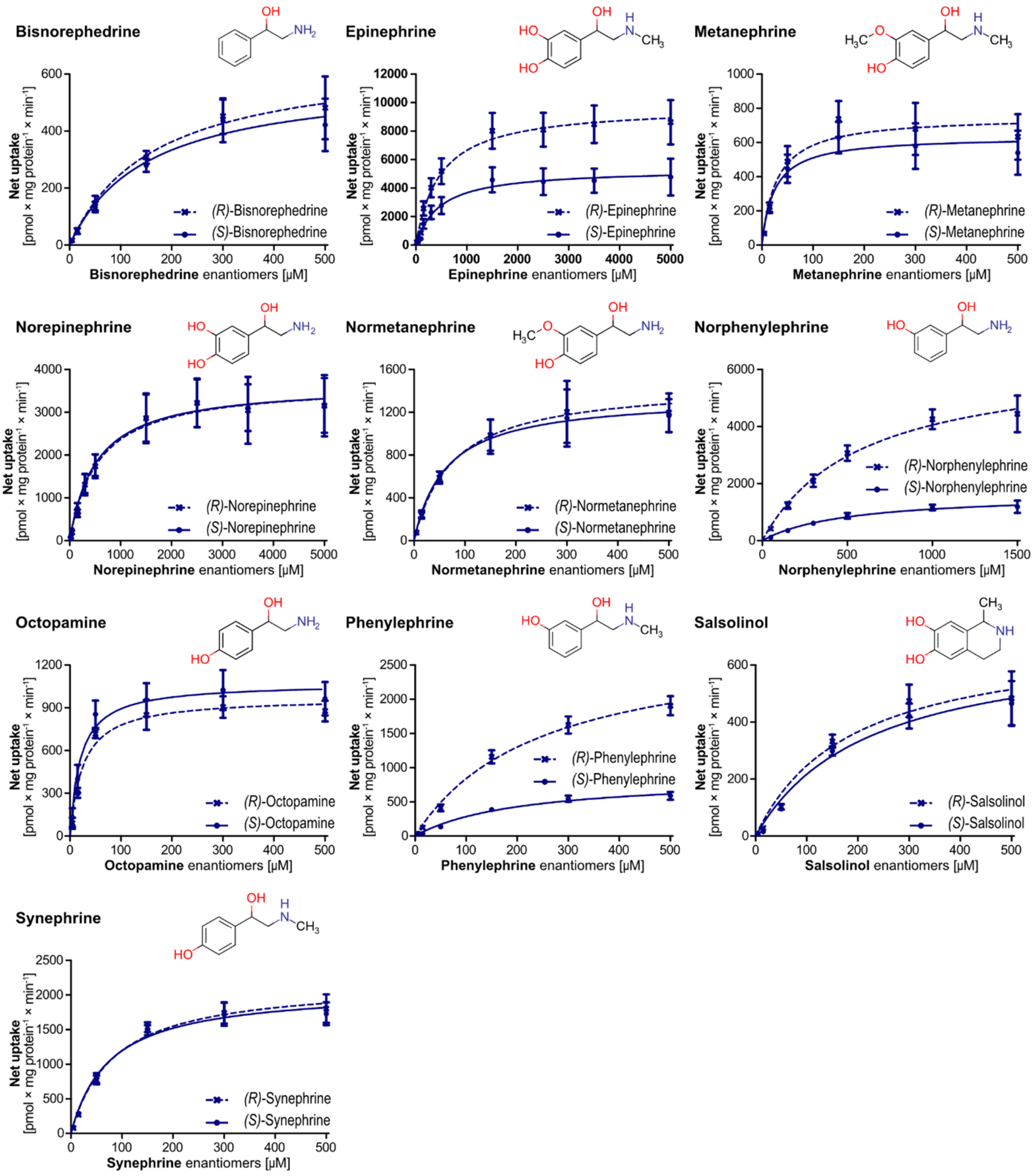

Figure S6 Net uptake curves of the organic cation transporter 3 (OCT3) for investigated chiral phenylethylamines (in alphabetical order). Data is presented as mean ± SEM of at least three independent experiments. Not transported substances (no net uptake) are not shown.

Table S1 HPLC conditions for chiral separation of investigated substances

**Substance**	**Mobile phase composition**	**Flow rate** [µL min^{-1}]	**Temperature** [°C]	**Retention time** [min; order of elution]
Bisnorephedrine	10 mM NH_4Ac, pH 5.8; 10% IPA	400	22	2.81 / 3.42 [R-S]
Epinephrine	10 mM NH_4Ac, pH 5.8; 10% IPA	300	22	3.83 / 4.83 [R-S]
Halostachine	10 mM NH_4Ac, pH 5.8; 10% IPA	300	22	3.04 / 3.38 [1-2]
Metanephrine	10 mM NH_4Ac, pH 5.8; 10% IPA	300	22	3.74 / 4.26 [1-2]
Norepinephrine	10 mM NH_4Ac, pH 5.8; 10% IPA	400	22	3.91 / 5.78 [R-S]
Normetanephrine	10 mM NH_4Ac, pH 5.8; 10% IPA	400	22	3.37 / 5.16 [1-2]
Norphenylephrine	10 mM NH_4Ac, pH 5.8; 10% IPA	400	22	3.30 / 4.33 [1-2]
Octopamine	10 mM NH_4Ac, pH 5.8; 10% IPA	400	22	3.31 / 5.36 [R-S]
Phenylephrine	10 mM NH_4Ac, pH 5.8; 10% IPA	400	22	2.65 / 3.07 [R-S]
Salsolinol	10 mM NH_4Ac, pH 5.0; 10% IPA	400	25	2.16 / 4.23 [1-2]
Synephrine	10 mM NH_4Ac, pH 5.8; 10% IPA	400	22	2.60 / 3.27 [R-S]

NH_4Ac, ammonium acetate; IPA, isopropyl alcohol

Table S2 Mass spectrometry detection parameters

Substance	Mass Q1[a] [Da]	Mass Q3[b] [Da]	DP[c] [V]	CE[d] [V]	CXP[e] [V]
Bisnorephedrine	137.890	119.910 (63.910)	29	12 (37)	8 (12)
Epinephrine	184.100	166.100 (135.000)	36	13 (15)	8 (10)
Halostachine	152.100	134.000 (119.000)	41	13 (27)	8 (8)
Metanephrine	198.100	165.000 (119.000)	41	25 (29)	8 (8)
Norepinephrine	170.200	152.000 (107.000)	36	10 (28)	10 (20)
Normetanephrine	184.100	166.000 (134.000)	36	9 (25)	10 (8)
Norphenylephrine	154.180	136.000 (91.100)	39	11 (29)	8 (16)
Octopamine	154.000	136.000 (91.000)	36	11 (29)	8 (16)
Phenylephrine	168.120	91.000 (77.000)	41	30 (56)	11 (4)
Salsolinol	180.080	117.100 (145.200)	81	31 (25)	8 (10)
Synephrine	167.691	149.900 (135.000)	36	35 (35)	12 (8)
Milnacipran	247.174	230.200 (100.100)	51	17 (27)	14 (8)

Detection parameters for a second substance-specific mass transition used as qualifier are shown in parentheses.

[a]Q1, first quadrupole

[b]Q3, third quadrupole

[c]DP, declustering potential

[d]CE, collision energy

[e]CXP, collision cell exit potential

Table S3 Kinetic parameters for the stereoselective transport of racemic monoamines by MATs and OCTs

Transporter	Substrate	K_m ± SEM [μM]	V_{max} ± SEM [pmol × mg protein^{-1} × min^{-1}]	Cl_{int} ± SEM [mL × g protein^{-1} × min^{-1}]	Stereoselectivity K_m	Stereoselectivity V_{max}	Stereoselectivity Cl_{int}
NET	*(R)*-Bisnorephedrine	0.206 ± 0.184	9.50 ± 1.39	46.1 ± 48.0	1.01-fold for *(R)*	1.23-fold for *(S)*	1.24-fold for *(S)*
	(S)-Bisnorephedrine	0.204 ± 0.157	11.7 ± 1.47	57.4 ± 51.3			
	Halostachine-1	No saturable net uptake					
	Halostachine-2						
	Norphenylephrine-1	2.25 ± 0.42	426 ± 13.1	189 ± 41.2	1.03-fold for 2	1.25-fold for 2**	1.21-fold for 2
	Norphenylephrine-2	2.31 ± 0.40	531 ± 15.1	230 ± 46.1			
	(R)-Phenylephrine	5.12 ± 0.95	392 ± 12.12	75.5 ± 16.2	1.03-fold for *(S)*	1.79-fold for *(R)****	1.43-fold for *(R)*
	(S)-Phenylephrine	4.16 ± 1.07	220 ± 9.26	52.3 ± 15.6			
	(R)-Octopamine	2.86 ± 0.35	476 ± 9.68	166 ± 23.4	1.55-fold for *(R)**	1.30-fold for *(R)****	1.19-fold for *(S)*
	(S)-Octopamine	1.85 ± 0.21	366 ± 6.91	198 ± 26.7			
	(R)-Synephrine	7.33 ± 2.86	273 ± 17.9	37.3 ± 17.0	2.61-fold for *(S)*	1.80-fold for *(R)***	4.70-fold for *(R)*
	(S)-Synephrine	19.1 ± 8.97	151 ± 13.4	7.92 ± 4.42			
	Normetanephrine-1	36.6 ± 13.5	99.4 ± 17.0	2.71 ± 1.47	1.35-fold for 1	1.06-fold for 1	1.28-fold for 2
	Normetanephrine-2	26.2 ± 9.53	94.2 ± 13.7	3.47 ± 1.72			
	Metanephrine-1	No saturable net uptake					
	Metanephrine-2						
	Salsolinol-1	90.1 ± 33.3	112 ± 20.1	1.24 ± 0.69	1.13-fold for 2	1.35-fold for 2	1.19-fold for 2
	Salsolinol-2	102 ± 35.8	151 ± 27.9	1.48 ± 0.79			
DAT	*(R)*-Bisnorephedrine	4.38 ± 2.91	27.2 ± 6.49	6.21 ± 5.61	1.18-fold for *(S)*	1.17-fold for *(S)*	1.01-fold for *(R)*
	(S)-Bisnorephedrine	5.16 ± 3.59	31.7 ± 8.4	6.15 ± 5.91			
	Halostachine-1	No saturable net uptake					
	Halostachine-2						
	Norphenylephrine-1	15.6 ± 2.47	2003 ± 64.5	128 ± 24.5	1.20-fold for 2	1.55-fold for 2***	1.29-fold for 2
	Norphenylephrine-2	18.6 ± 2.99	3103 ± 104	167 ± 32.3			
	(R)-Phenylephrine	35.6 ± 6.13	613 ± 25.2	17.2 ± 3.68	1.06-fold for *(R)*	1.56-fold for *(R)****	1.48-fold for *(R)*
	(S)-Phenylephrine	33.7 ± 8.37	392 ± 22.9	11.6 ± 3.57			
	(R)-Octopamine	20.5 ± 3.22	2054 ± 68.2	100 ± 19.1	1.05-fold for *(R)*	1.05-fold for *(R)*	1.00-fold for *(R/S)*
	(S)-Octopamine	19.6 ± 4.06	1959 ± 85.2	100 ± 25.0			

Transporter	Substrate	K_m ± SEM [µM]	V_{max} ± SEM [pmol × mg protein^{-1} × min^{-1}]	Cl_{int} ± SEM [mL × g protein^{-1} × min^{-1}]	Stereoselectivity K_m	Stereoselectivity V_{max}	Stereoselectivity Cl_{Int}
**DAT**	*(R)*-Synephrine	36.8 ± 6.85	849 ± 34.0	23.1 ± 5.22	1.21-fold for *(S)*	1.84-fold for *(R)******	2.23-fold for *(R)*
	(S)-Synephrine	44.7 ± 9.23	463 ± 21.8	10.4 ± 2.63			
	Normetanephrine-1	No saturable net uptake					
	Normetanephrine-2						
	Metanephrine-1	No saturable net uptake					
	Metanephrine-2						
	Salsolinol-1	No saturable net uptake					
	Salsolinol-2						
**SERT**	*(R)*-Bisnorephedrine	No saturable net uptake					
	(S)-Bisnorephedrine						
	Halostachine-1	No saturable net uptake					
	Halostachine-2						
	Norphenylephrine-1	333 ± 106	400 ± 64.9	1.20 ± 0.58	1.20-fold for 1	1.27-fold for 2	1.53-fold for 2
	Norphenylephrine-2	277 ± 68.7	508 ± 59.5	1.83 ± 0.67			
	(R)-Phenylephrine	328 ± 106	356 ± 59.4	17.2 ± 3.68	1.89-fold for *(R)*	1.49-fold for *(S)**	2.82-fold for *(S)**
	(S)-Phenylephrine	173 ± 20.6	529 ± 26.5	11.6 ± 3.57			
	(R)-Octopamine	59.6 ± 11.3	247 ± 12.6	4.14 ± 1.00	1.39-fold for *(R)*	2.38-fold for *(R)******	1.71-fold for *(R)*
	(S)-Octopamine	42.9 ± 14.2	104 ± 8.39	2.42 ± 1.00			
	(R)-Synephrine	528 ± 440	363 ± 182	0.69 ± 0.92	1.52-fold for *(S)*	1.35-fold for *(S)*	1.13-fold for *(R)*
	(S)-Synephrine	804 ± 868	491 ± 364	0.61 ± 1.11			
	Normetanephrine-1	42.0 ± 12.5	93.6 ± 13.4	2.23 ± 0.98	1.08-fold for 1	1.67-fold for 2*	1.28-fold for 2
	Normetanephrine-2	38.7 ± 6.62	157 ± 12.5	4.05 ± 1.01			
	Metanephrine-1	151 ± 165	87.4 ± 57.3	0.58 ± 1.01	1.09-fold for 1	1.08-fold for 2	1.01-fold for 1
	Metanephrine-2	165 ± 152	94.5 ± 53.6	0.57 ± 0.85			
	Salsolinol-1	129 ± 26.0	229 ± 23.4	1.77 ± 0.54	1.85-fold for 2	1.34-fold for 1	2.48-fold for 1
	Salsolinol-2	239 ± 65.6	171 ± 29.0	0.71 ± 0.32			
**OCT1**	*(R)*-Bisnorephedrine	187 ± 151	218 ± 71.7	1.17 ± 1.33	1.15-for for *(R)*	1.19-fold for *(R)*	1.03-fold for *(R)*
	(S)-Bisnorephedrine	162 ± 130	184 ± 56.4	1.13 1.25			
	Halostachine-1	No saturable net uptake					
	Halostachine-2						

Transporter	Substrate	K_m ± SEM [µM]	V_{max} ± SEM [pmol × mg protein^{-1} × min^{-1}]	Cl_{int} ± SEM [mL × g protein^{-1} × min^{-1}]	Stereoselectivity		
					K_m	V_{max}	Cl_{int}
OCT1	Norphenylephrine-1	175 ± 46.0	1710 ± 177	4.02 ± 1.76	1.03-fold for 1	1.08-fold for 1	1.05-fold for 1
	Norphenylephrine-2	178 ± 49.1	1578 ± 172	3.84 ± 1.75			
	(R)-Phenylephrine	175 ± 46.0	1710 ± 177	9.79 ± 3.59	1.02-fold for *(S)*	1.08-fold for *(R)*	1.11-fold for *(R)*
	(S)-Phenylephrine	178 ± 49.1	1578 ± 172	8.85 ± 3.40			
	(R)-Octopamine	291 ± 56.6	1318 ± 85.3	4.53 ± 1.17	1.04-fold for *(R)*	1.09-fold for *(R)*	1.05-fold for *(R)*
	(S)-Octopamine	281 ± 61.2	1211 ± 86.7	4.32 ± 1.25			
	(R)-Synephrine	86.7 ± 11.0	1817 ± 65.7	21.0 ± 3.42	1.28-fold for *(S)*	1.35-fold for *(R)***	1.73-fold for *(R)*
	(S)-Synephrine	111 ± 18.4	1348 ± 70.5	12.1 ± 2.65			
	Normetanephrine-1	1279 ± 869	3145 ± 1530	2.46 ± 2.87	1.59-fold for 1	1.47-fold for 1	1.08-fold for 2
	Normetanephrine-2	806 ± 394	2137 ± 669	2.65 ± 2.13			
	Metanephrine-1	134 ± 48.4	1082 ± 139	8.09 ± 3.96	1.09-fold for 2	1.06-fold for 1	1.16-fold for 1
	Metanephrine-2	146 ± 50.9	1018 ±	6.97 ± 3.32			
	Salsolinol-1	133 ± 68.3	1450 ± 429	10.9 ± 8.84	1.02-fold for 1	1.02-fold for 1	1.00-fold for 1/2
	Salsolinol-2	131 ± 71.2	1426 ± 444	10.9 ± 9.4			
OCT2	*(R)*-Bisnorephedrine	37.5 ± 11.4	847 ± 60.9	22.6 ± 8.50	1.61-fold for *(S)*	1.41-fold for *(R)**	2.27-fold for *(R)*
	(S)-Bisnorephedrine	60.4 ± 18.2	602 ± 48.8	10.0 ± 3.81			
	Halostachine-1	12.8 ± 14.3	329 ± 70.0	25.7 ± 34.0	1.50-fold for *(S)*	1.26-fold for *(R)*	1.89-fold for *(R)*
	Halostachine-2	19.3 ± 23.7	261 ± 66.3	13.6 ± 20.2			
	Norphenylephrine-1	911 ± 88.7	33143 ± 1628	36.4 ± 5.33	1.57-fold for 1*	2.19-fold for 1***	1.40-fold for 1
	Norphenylephrine-2	582 ± 63.7	15157 ± 712	26.07 ± 4.08			
	(R)-Phenylephrine	212 ± 72.9	6263 ± 914	29.5 ± 11.4	1.89-fold for *(R)*	2.04-fold for *(R)**	1.02-fold for *(S)*
	(S)-Phenylephrine	112 ± 36.7	3068 ± 332	27.3 ± 11.9			
	(R)-Octopamine	636 ± 83.6	25182 ± 1468	39.6 ± 7.52	1.63-fold for *(R)**	2.14-fold for *(R)****	1.31-fold for *(R)*
	(S)-Octopamine	281 ± 61.2	11762 ± 378	30.2 ± 3.6			
	(R)-Synephrine	443 ± 154	10877 ± 2130	24.6 ± 13.3	2.07-fold for *(R)*	2.05-fold for *(R)*	1.01-fold for *(S)*
	(S)-Synephrine	214 ± 76.2	5307 ± 807	24.8 ± 12.6			
	Normetanephrine-1	613 ± 93.9	5665 ± 491	9.24 ± 2.22	1.32-fold for 1	2.06-fold for 2*	1.56-fold for 1
	Normetanephrine-2	464 ± 227	2747 ± 686	5.92 ± 4.37			
	Metanephrine-1	54.7 ± 22.0	1127 ± 118	20.6 ± 10.4	1.25-fold for 2	2.13-fold for 1*	2.66-fold for 1
	Metanephrine-2	68.1 ± 30.7	528 ± 66.3	7.75 ± 4.46			

Transporter	Substrate	K_m ± SEM [µM]	V_{max} ± SEM [pmol × mg protein^{-1} × min^{-1}]	Cl_{int} ± SEM [mL × g protein^{-1} × min^{-1}]	Stereoselectivity		
					K_m	V_{max}	Cl_{int}
**OCT2**	Salsolinol-1	222 ± 93.6	9083 ± 1659	40.9 ± 24.7	1.04-fold for 1	1.01-fold for 2	1.05-fold for 2
	Salsolinol-2	213 ± 89.3	9160 ± 1634	42.7 ± 25.6			
**OCT3**	*(R)*-Bisnorephedrine	165 ± 83.3	662 ± 126	4.00 ± 2.77	1.08-fold for *(R)*	1.13-fold for *(R)*	1.04-fold for *(R)*
	(S)-Bisnorephedrine	153 ± 78.4	589 ± 111	3.85 ± 2.70			
	Halostachine-1	No saturable net uptake					
	Halostachine-2						
	Norphenylephrine-1	582 ± 162	6412 ± 767	11.0 ± 4.39	1.17-fold for 1	3.88-fold for 1**	3.32-fold for 1
	Norphenylephrine-2	498 ± 165	1654 ± 222	3.32 ± 1.54			
	(R)-Phenylephrine	244 ± 56.9	2885 ± 303	11.8 ± 3.99	1.19-fold for *(R)*	3.33-fold for *(R)****	2.79-fold for *(R)*
	(S)-Phenylephrine	205 ± 56.1	866 ± 99.5	4.23 ± 1.64			
	(R)-Octopamine	25.1 ± 7.01	972 ± 54.2	38.8 ± 13.0	1.38-fold for *(S)*	1.10-fold for *(S)*	1.38-fold for *(S)*
	(S)-Octopamine	20.1 ± 7.68	1072 ± 77.1	53.4 ± 24.2			
	(R)-Synephrine	83.5 ± 19.5	2193 ± 153	26.3 ± 8.00	1.11-fold for *(R)*	1.05-fold for *(R)*	1.06-fold for *(S)*
	(S)-Synephrine	75.3 ± 16.7	2090 ± 133	27.8 ± 7.93			
	Normetanephrine-1	75.9 ± 32.7	1473 ± 175	19.4 ± 10.7	1.22-fold for 1	1.09-fold for 1	1.12-fold for 2
	Normetanephrine-2	62.4 ± 27.4	1352 ± 161	21.6 ± 12.1			
	Metanephrine-1	29.0 ± 13.9	752 ± 80.6	25.9 ± 15.2	1.19-fold for 1	1.19-fold for 1	1.00-fold for 1/2
	Metanephrine-2	24.4 ± 12.3	635 ± 68.6	26.0 ± 15.8			
	Salsolinol-1	186 ± 88.8	705 ± 133	3.80 ± 2.52	1.20-fold for 2	1.01-fold for 1	1.21-fold for 1
	Salsolinol-2	222 ± 90.9	697 ± 122	3.15 ± 1.84			

SEM, standard error of the mean; asterisks indicate statistical significance of the differences between the two enantiomers (Student's *t*-test; *$p < 0.05$, **$p < 0.01$, ***$p < 0.001$).

Table S4 Stereoselectivity and transporter selectivity of investigated phenylethylamines for OCT2 and its A270S variant

Substrate	Stereoselectivity OCT2_WT	Stereoselectivity OCT2_A270S	Transporter selectivity	
Bisnorephedrine	1.10-fold for *(R)*	1.09-fold for *(S)*	*(R)*-Bisnorephedrine	1.50-fold for WT
			(S)-Bisnorephedrine	1.24-fold for WT
Epinephrine	1.81-fold for *(R)**	1.93-fold for *(R)****	*(R)*-Epinephrine	1.19-fold for WT
			(S)-Epinephrine	1.19-fold for WT
Halostachine	1.42-fold for *(R)*	1.32-fold for *(R)*	*(R)*-Halostachine	1.02-fold for WT
			(S)-Halostachine	1.05-fold for A270S
Metanephrine	2.78-fold for *(R)****	2.26-fold for *(R)****	*(R)*-Metanephrine	1.20-fold for A270S
			(S)-Metanephrine	1.47-fold for A270S
Norepinephrine	2.05-fold for *(R)**	2.06-fold for *(R)**	*(R)*-Norepinephrine	1.21-fold for WT
			(S)-Norepinephrine	1.22-fold for WT
Normetanephrine	1.91-fold for *(R)***	1.70-fold for *(R)***	*(R)*-Normetanpehrine	1.21-fold for WT
			(S)-Normetanephrine	1.08-fold for WT
Norphenylephrine	1.54-fold for *(R)****	1.46-fold for *(R)****	*(R)*-Norphenylephrine	1.18-fold for WT**
			(S)-Norphenylephrine	1.12-fold for WT*
Octopamine	1.74-fold for *(R)***	1.69-fold for *(R)***	*(R)*-Octopamine	1.20-fold for WT
			(S)-Octopamine	1.17-fold for WT
Phenylephrine	1.30-fold for *(R)**	1.16-fold for *(R)*	*(R)*-Phenylephrine	1.27-fold for WT*
			(S)-Phenylephrine	1.13-fold for WT
Salsolinol	1.06-fold for *(S)*	1.10-fold for *(S)*	*(R)*-Salsolinol	1.50-fold for WT*
			(S)-Salsolinol	1.45-fold for WT**
Synephrine	1.17-fold for *(R)*	1.00-fold for *(R)*	*(R)*-Synephrine	1.41-fold for WT*
			(S)-Synephrine	1.20-fold for WT

Asterisks indicate statistical significance of the differences between the two enantiomers or between the uptake by OCT2 wildtype compared to the uptake by the OCT2 A270S variant (Student's *t*-test; *p < 0.05, **p < 0.01, ***p < 0.001).
